# Correction to Vascular flow void signal in pituitary neuroendocrine tumors: A case report and review of literature

**DOI:** 10.1002/ccr3.9081

**Published:** 2024-06-11

Funding information needs to be updated.

Zhong J, Li J, Wang S. Vascular flow empty shadow in pituitary neuroendocrine tumors: A case report and review of literature. *Clin Case Rep*. 2023;11(10):e7931. doi:10.1002/ccr3.7931

In the Acknowledgment section, information on the Foundation's projects is missing. Replace “We thank the Neurosurgery Department of Fuzong Clinical Medical College of Fujian Medical University. The present study was funded by the Fujian Provincial Key Project of Science and Technology Plan (Grant No. 2019Y9045) and the Fujian Medical University Sailing Fund Project (grant no. 2019QH2043)” with “We thank the Neurosurgery Department of Fuzong Clinical Medical College of Fujian Medical University. This study was supported by the Key Project of Science and Technology Plan of Fujian Province (Grant No. 2019Y9045), the Yangfan Fund Project of Fujian Medical University (Grant No. 2019QH2043), and the Joint Logistics Medical Key Speciality Project (Grant No. LQZD‐SW)” in the Acknowledgment section of the text.

We apologize for this error.

## AUTHOR CONTRIBUTIONS

**Jiansheng Zhong:** Writing – original draft. **Jun Li:** Writing – review and editing. **Shousen Wang:** Funding acquisition; writing – review and editing.

## Data Availability

The data that support the findings of this study are available on request from the corresponding author (Shousen Wang), upon reasonable request.

